# Evaluating the accuracy, reliability, and agreement of the İşcan and Hartnett age estimation methods on a contemporary European skeletal sample of fourth ribs

**DOI:** 10.1093/fsr/owaf021

**Published:** 2025-08-22

**Authors:** Gian Luca Marella, Giulia Ceccobelli, Claudia Reitano, Alessandro Mauro Tavone, Raimondo Vella, Gabriele Giuga, Antonio Vinci, Fabio Ingravalle, Saverio Potenza, Massimo Maurici, Maria Cristina Martinez-Labarga

**Affiliations:** Department of Surgical Sciences, Tor Vergata University of Rome, Rome, Italy; Department of Biomedicine and Prevention, Tor Vergata University of Rome, Rome, Italy; Department of Biology, Tor Vergata University of Rome, Rome, Italy; Department of Surgical Sciences, Tor Vergata University of Rome, Rome, Italy; Department of Biomedicine and Prevention, Tor Vergata University of Rome, Rome, Italy; Department of Biomedicine and Prevention, Tor Vergata University of Rome, Rome, Italy; Department of Biomedicine and Prevention, Tor Vergata University of Rome, Rome, Italy; Department of Biomedicine and Prevention, Tor Vergata University of Rome, Rome, Italy; Department of Biomedicine and Prevention, Tor Vergata University of Rome, Rome, Italy; Department of Biomedicine and Prevention, Tor Vergata University of Rome, Rome, Italy; Department of Biology, Tor Vergata University of Rome, Rome, Italy

**Keywords:** forensic sciences, forensic anthropology, age estimation, human identifications, rib analysis, reproducibility of results

## Abstract

This study evaluates the accuracy and reliability of the İşcan and Hartnett methods for estimating the age of adults based on rib analysis, using a sample of 127 pairs of ribs from a contemporary European population. The study employed a double-blind design with repeated measurements conducted by two observers. The İşcan method demonstrated a higher success rate, accurately assigning age in 62% of cases, compared to 38% for the Hartnett method. Both methods exhibited moderate intra- and interoperator agreement, as measured by Cohen’s Kappa. A detailed statistical analysis, including logistic regression, revealed significant discrepancies in phase-assignment accuracy between the two methods. The İşcan method’s success rate improved when prioritizing the highest observed phase, indicating potential for enhancing accuracy through strategic methodological adjustments. The findings underscore the importance of operator training and the need for consistent application of criteria. This research highlights the critical need for standardization in age estimation methods and suggests potential improvements for forensic and anthropological applications. The study contributes valuable insights into the strengths and limitations of widely used skeletal age estimation techniques, with implications for improving methodological consistency and accuracy in forensic investigations.

**Key points**
 

- Human age can be estimated through the study of the fourth rib in skeletal remains; the two most commonly used methods are those of İşcan and Hartnett.
- Both the İşcan and Hartnett methods showed moderate inter- and intraoperator agreement.
- The İşcan method exhibited significant overlap in age ranges, particularly in later phases, which can lead to underestimation of age.
- The findings emphasize the importance of extensive training for operators using these methods.

## Introduction

The assessment of the age of an individual is one of the main challenges of forensic anthropology, playing a fundamental role both in identification of skeletal remains and in living subjects. In most cases the age assessment of skeletal remains is carried out in the laboratory after careful preparation and execution of particular investigations, some traditional ones based on morphological examination and others instrumental through radiographic and histological examination [1].

The choice of the appropriate methodology is dependent on the general pattern of preservation of the remains and the specificity of the case [2]. Age estimation is generally more accurate in growing individuals [3].

As noted by Scheuer [4], the skeleton in children and young adults undergoes changes that follow a predictable and well-documented pattern. After growth, these changes become more varied, even within the same skeleton, and become more individual and population-specific. In younger individuals, age can be estimated by evaluating the eruption of teeth, their degree of mineralization and the development of ossification centres. In young adults, the bone segments that are still ossifying between the second and third decades of life are usually analysed: the spheno-occipital synchondrosis, the occipitomastoid suture, the bodies of the sacral vertebrae, the spine, the scapula, the rib incisions of the sternum, and the medial portion of the clavicle. Finally, in adults the most traditionally used methods are analysis of the state of fusion of the cranial sutures and variations in the pubic symphysis and sacroiliac joints. However, in 1984 İşcan et al. [5] proposed a new method for estimating the age at death in adults based on morphological study of the sternal end of the fourth rib. The choice of the fourth rib was purely conventional, this being a bone fragment easily found during a routine autopsy.

Initially, a sample of 118 specimens of the fourth rib from male subjects was analysed; all the specimens came from individuals of known age, sex, and geographical origin [6]. Morphological analysis showed that the first changes in the sternal articular surface of the fourth male rib occur after the age of 16; therefore, all subjects aged 16 or younger were excluded. Subsequently, the bone fragments were divided into nine groups corresponding to different phases (from 0 to 8), with specific age ranges associated with each phase. Phasing was carried out based on the observed changes in the shape, structure, and overall quality of the sternal end of the rib.

The same approach was later used by the same authors on a sample of 86 ribs of female subjects [7]. In this case, it was noted that the changes were evident from the age of 14, so nine new phases for the female sex were established. İşcan himself confirmed the existence of a sexual dimorphism in the ribs, stressing, moreover, how it becomes more evident with increasing age [8].

Sanders [9] had already made the same observation almost 20 years earlier, noting that women undergoing hysterectomy or oophorectomy had a mineralization pattern of costal cartilage more similar to that of men. These results were predictable given the known difference in hormone production between males and females and the pronounced sexual dimorphism in other parts of the skeleton [7].

The same observations were confirmed by more recent studies, which were also carried out using the most modern imaging techniques [10–12].

In a 2010 study, Hartnett [13] applied the İşcan method to a larger sample of 630 specimens to assess its accuracy and found that the method was quite reliable but needed some refinement. The most important innovation introduced by Hartnett was represented by the evaluation of bone quality and density while maintaining the assessment of morphological aspects of the articular surface, such as the depth of the pit and the regularity of the edges. This significant innovation allowed the definition of a new distribution in eight phases (from 1 to 7), defined differently from previous studies. Such phases had narrower age ranges than those proposed by İşcan and no distinction between sexes.

The objective of the present study is to evaluate the relative accuracy rate, reliability, and agreement level of the İşcan and Hartnett age estimation methods on a contemporary European skeletal sample.

## Materials and methods

### Design, population, and data sources

This study was designed as a double-blind reliability and agreement study. A convenience sample was used, consisting of all available fourth rib pairs within the Institute of Legal Medicine of the University of Rome “Tor Vergata”, Italy, from July 2017 to September 2022. The investigated sample consisted of a set of 144 pairs of ribs. Only specimens from people of European origin and of known age at the time of death were included in the final analysis.

Before proceeding to the collection of samples, a formal request was made to the Independent Ethics Committee of the Policlinico Tor Vergata, which granted the authorization for the collection of the bones of interest in the autopsies at the Tor Vergata Hospital (prot. 71/17).

The collection of the fragments was performed bilaterally through a section carried out 3 cm medially and laterally to the costochondral joint, during the autoptic examination. The samples were anonymized and recorded in a record sheet showing the collection identification number, the date and location of the autopsy, and the histology identification number and information on the cadaver such as sex, age, height, and geographical ancestry. After sampling, the ribs were stored in a container containing a solution of soap and water in order to facilitate the maceration of soft tissues still attached to the skeletal segments and the melting of fats. The storage period within this solution was 2 weeks, after which the fragments could be cleaned. After that, the fourth rib fragments were boiled under an extractor hood for 10–15 min in order to further facilitate the detachment of soft tissues. Subsequently, with the aid of a surgical scalpel and tweezers, the soft tissues still attached and the costal cartilage present inside the articular facet of the rib were removed. Finally, the clean bone fragments were left to air dry on sheets of tissue paper for about 20 d.

After this period, using the study protocols of İşcan and Hartnett, it was possible to carry out the morphological examination of each pair of fourth ribs.

For each specimen, data on sex, age at death, and geographical origin (Caucasian population *vs.* others) were collected from medical records and stored in a separate dataset for subsequent analysis. A simple numerical ID was then used for pseudoanonymization, ensuring that each specimen was identifiable with information on sex but no information on age.

### Sampling and evaluation procedure

We chose by design to include observers with the same experience level (formal and practical training in forensic pathology and anthropology, with little previous experience in rib classification), to avoid any bias due to different expertise between observers and to guarantee the generalizability of our results. The two operators performed an independent evaluation on each individual’s fourth rib. Each operator independently assigned both İşcan and Hartnett phases to each rib. Each rib was measured five times by each operator at intervals of at least 14 d to avoid memorization bias. Measures were repeated in random order, and operators had no access to previous measurement data, clinical information, or any recorded details about the specimen.

### Statistical analysis and reporting

#### Descriptive analysis

For each phase, the actual specimen age distribution was described by the mean and standard deviation (SD), as observed by each operator, separately for male and female subjects. The Shapiro–Wilk test was used to check the normal distribution of data. A *P*-value ≤ 0.05 was considered significant. The observed phase results were plotted, differentiated by operator, classification, and sex. Estimation error between observed and actual age (underestimation or overestimation) was analysed by sex and age using the Epanechnikov kernel density estimation (KDE) quadratic function [14].

#### Agreement and reliability

Inter- and intraoperator agreement was measured using Cohen’s Kappa [15].

For each observation, “success” was defined as assigning a phase compatible with actual age at death. For each rib, an operator’s success rate was calculated as the number of successes divided by the number of evaluations—5 evaluations for each observer. This was plotted to obtain a success rate curve, modelled as a classical logistic function. Differences in each operator’s success rate were then investigated using two-sample Kolmogorov–Smirnov tests for equality of distributions [16].

For each phase, prior probability was computed for each rib, defined as:


$$ {\mathrm{Prior}}_{\mathrm{phase}}=\left\{\begin{array}{l}0,\mathrm{age}\notin \left[{\operatorname{Min}}_{\mathrm{phase}},{\operatorname{Max}}_{\mathrm{phase}}\right]{}\ \\{}\ \\{}\frac{1}{\sum \mathrm{compatible}\ \mathrm{phases}},\mathrm{age}\in \left[{\operatorname{Min}}_{\mathrm{phase}},{\operatorname{Max}}_{\mathrm{phase}}\right]\ \end{array}\right. $$

In other words, a rib of a 27-year-old female subject would have a prior probability of 0 for phases I, IV, V, VI, and VII of Hartnett classification and a prior probability of 0.5 for phases II and III.

Posterior probability was instead defined as the number of times a rib was assigned to each phase, divided by the total number of observations. For each phase, prior and posterior probability densities were then computed and plotted. This comparison of observed and expected assignation for each phase allows for the identification of under- and overassignation.

For each observation, we computed whether the estimated age was over- or underestimating the actual age. We then investigated, by specimen age and sex, the average tendency of under- or overestimating the actual age for each method.

#### Sensitivity analysis and reporting

In order to investigate alternate strategies for improving success rates in dubious phase assignment scenarios, a sensitivity analysis was conducted by computing success rates for single-evaluation scenarios alternately using the lowest, modal, and maximum phases assigned to each rib.

The Guidelines for Reporting Reliability and Agreement Studies were used for reporting the results of this study [17]. Statistical significance was set at *P* = 0.05 for all inferential analyses.

## Results

### Descriptive analysis

The collected sample consisted of 144 pairs of ribs, collected at the Institute of Legal Medicine of the University of Rome “Tor Vergata”, Italy, from July 2017 to September 2022.

Seventeen specimens (11.8%) were excluded: 12 because of missing data on actual age at death and five because of non-European origin. The investigated specimens’ sample (*n* = 127) consisted of 88 male and 39 female subjects. The mean age was 53.6 ± 16.1 years for males and 59.2 ± 22.4 years for females. The sample distribution, by phase, is detailed in Table 1; several ribs could correspond to many phases, especially for İşcan classification, as its age ranges are notoriously wider and overlapping compared to Hartnett.

**Table 1:** Actual rib age for each observed phase, by sex, classification type, and operator.

Sex (method)	Phase	*N* of ribs (compatible within phase)	Expected *n* by each OP	OP 1			OP 2		
				Observed *n*	Mean ± SD (years)	Median (IQR) (years)	Observed *n*	Mean ± SD (years)	Median (IQR) (years)
Female (İşcan)	I	0	0	0	–	–	0	–	–
	II	2	5	9	20.55 ± 4.21	17.00 (17.00, 25.00)	3	19.67 ± 4.62	17.00 (17.00, 21.00)
	III	1	2	18	25.94 ± 6.16	25.00 (22.00, 30.00)	18	22.44 ± 4.90	22.00 (17.00, 25.00)
	IV	6	15	11	32.09 ± 14.49	25.00 (25.00, 38.00)	18	35.56 ± 15.64	30.00 (25.00, 42.00)
	V	24	60	65	61.22 ± 16.46	60.00 (47.00, 76.00)	52	60.88 ± 15.99	60.00 (48.00, 77.00)
	VI	24	60	65	71.29 ± 15.10	76.00 (59.00, 82.00)	73	69.00 ± 16.25	74.00 (56.00, 80.00)
	VII	22	55	27	71.92 ± 13.97	73.00 (57.00, 83.00)	27	71.04 ± 15.60	73.00 (55.00, 84.00)
	VIII	18	45	0	–	–	4	84.00 ± 2.00	83.00 (83.00, 84.00)
Male (İşcan)	I	1	3	0	–	–	0	–	–
	II	3	9	8	21.25 ± 1.39	22.00 (21.00, 22.00)	8	21.63 ± 0.52	22.00 (21.00, 22.00)
	III	11	32	35	27.45 ± 5.13	29.00 (26.00, 30.00)	36	28.86 ± 8.47	29.00 (26.00, 30.00)
	IV	12	35	74	47.55 ± 13.01	44.00 (39.25, 59.50)	84	48.12 ± 13.80	47.00 (38.00, 58.00)
	V	33	96	152	54.79 ± 11.83	53.00 (48.00, 65.00)	138	55.43 ± 11.08	53.00 (49.25, 64.50)
	VI	66	192	101	57.33 ± 9.91	54.00 (51.00, 63.00)	127	58.91 ± 12.42	56.00 (51.00, 67.50)
	VII	65	189	52	66.27 ± 12.51	65.00 (57.00, 77.75)	41	70.00 ± 14.41	72.00 (57.00, 83.00)
	VIII	65	189	18	78.83 ± 13.50	83.50 (64.25, 90.75)	6	63.17 ± 10.21	59.00 (59.00, 59.00)
Female (Hartnett)	I	0	0	0	–	–	0	–	–
	II	1	7	10	20.20 ± 4.13	21.00 (17.00, 25.00)	7	20.71 ± 3.68	22.00 (17.00, 23.50)
	III	2	13	21	26.86 ± 6.72	25.00 (22.00, 30.00)	19	24.53 ± 6.34	25.00 (19.50, 27.50)
	IV	3	20	22	45.09 ± 14.88	42.00 (42.00, 51.00)	27	46.89 ± 18.00	48.00 (34.00, 58.00)
	V	5	33	92	67.05 ± 16.34	71.50 (56.00, 82.00)	72	65.90 ± 16.71	71.50 (54.75, 79.00)
	VI	7	46	45	71.84 ± 13.7	74.00 (60.00, 83.00)	58	67.97 ± 15.65	73.00 (55.00, 80.00)
	VII	6	39	5	80.00 ± 12.45	83.00 (80.00, 87.00)	12	83.00 ± 7.90	83.00 (83.00, 87.00)
Male (Hartnett)	I	3	13	0	–	–	0	–	–
	II	4	17	15	22.33 ± 3.33	22.00 (21.00, 24.00)	9	22.22 ± 3.19	22.00 (21.00, 22.00)
	III	10	42	32	30.47 ± 8.31	29.00 (28.50, 30.00)	30	29.27 ± 11.21	29.00 (26.25, 30.00)
	IV	14	59	146	49.92 ± 12.86	48.50 (40.00, 59.00)	131	48.75 ± 13.25	48.00 (39.00, 59.00)
	V	34	144	137	57.19 ± 9.65	54.00 (51.00, 63.00)	127	57.65 ± 10.49	54.00 (51.00, 67.00)
	VI	22	93	91	63.08 ± 12.67	60.00 (54.00, 77.00)	127	61.21 ± 14.66	58.00 (51.00, 72.00)
	VII	17	72	19	76.95 ± 15.48	83.00 (59.00, 88.50)	16	67.25 ± 11.91	65.00 (59.00, 77.75)

SD: standard deviation; OP: operator; IQR: interquartile range.

Two observers (G.C. and C.R.) observed five times each of the 127 pairs of ribs, for a total of 635 observations. No difference emerged in left and right rib class assignation.

The Shapiro–Wilk test confirmed the skewed distribution of phase scores across the sample (*P* < 0.001), as well as for both operators: OP 1 (*P* = 0.048) and OP 2 (*P* = 0.008). For each classification and phase, the mean and SD are reported in Table 1. No significant difference was found in the age ranges.

### Agreement and reliability

For İşcan classification, intraoperator agreement was found to be moderate for both operators, with Cohen’s Kappa values of 0.53 (OP 1) and 0.49 (OP 2); interoperator agreement was also moderate (Kappa = 0.47). Similarly, intraoperator agreement for Hartnett classification was moderate, with Cohen’s Kappa values of 0.54 (OP 1) and 0.51 (OP 2), and a value of 0.49 for interoperator agreement. The *P*-values were < 0.001 for all estimations.

**Figure 1:**
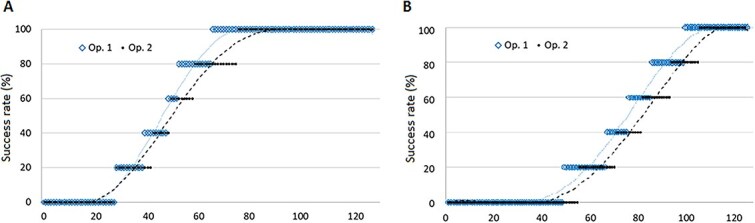
Success rate, by method and operator. (A) İşcan success rate; (B) Hartnett success rate.

The İşcan assignment success rate was found to be 62%, with a slight, nonsignificant difference in success rate between operators (*P* = 0.06). The Hartnett assignment success rate was much lower, 38%, again with no significant difference between operators (*P* = 0.826). The overall success rates for the two methods are depicted in Figure 1.

**Figure 2:**
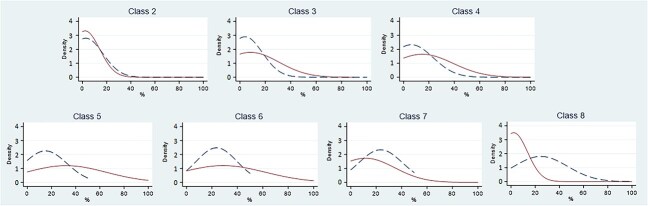
Prior and posterior density distribution for each İşcan phase. Prior: dashed line. Posterior: solid line. Class 1 omitted because no observations available.

**Figure 3:**
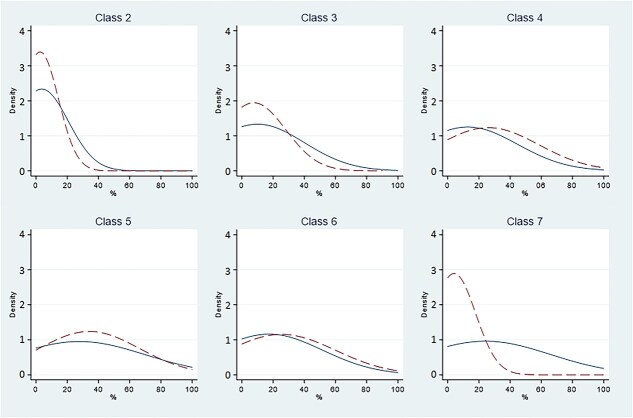
Prior and posterior density distribution for each Hartnett phase. Prior: dashed line. Posterior: solid line. Class 1 omitted because no observations available.

Prior and posterior distribution densities are depicted in Figures 2 and 3. For the İşcan methodology, prior probability was overrepresented in higher classes (V or higher). For the Hartnett method, prior probability was overrepresented for the most extreme classes only (II and VII). The difference between prior and posterior probability distribution is overall more marked in the İşcan classification, and differences are more prominent for the most extreme phases for both methods.

**Figure 4:**
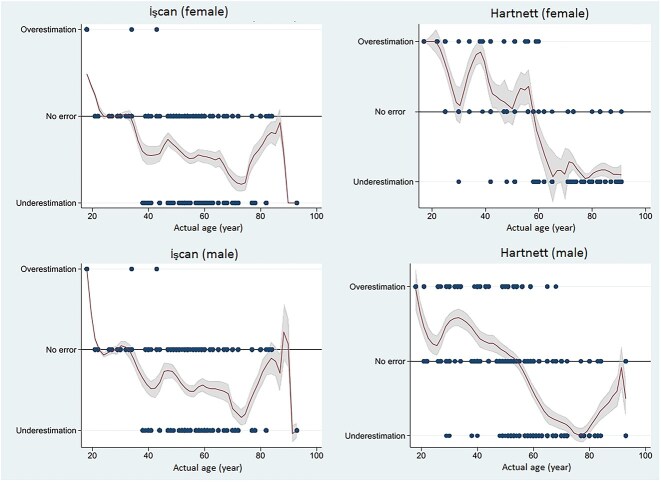
Epanechnikov kernel density estimation (KDE) for under/overestimation of the age in the two methods.

To highlight differences in accuracy, we investigated the tendency to under- or overestimate, by sex, according to the specimen age. We found that both methods tend to underestimate actual age (assigning a class lower than expected). The İşcan method was closer to the actual estimation, and never overestimated when the actual age was >40 years. The Hartnett method was less reliable and systematically underestimated when the actual age was >70 years. A depiction of each method’s misclassification tendency, distinct by sex, is available in Figure 4.

### Sensitivity analysis: success maximization

For both classification systems, there is no literature on the optimal number of measures to take when evaluating a rib phase. In this study, each operator evaluated the same rib five times, but ideally, a single evaluation should be performed. Since in several instances the same rib was evaluated differently, even by the same operator, we assumed during the analytical phase that some assignments could be borderline or dubious. The operators had no heuristic to follow in such cases. For this reason, we evaluated the phase assignment success rate under three single-evaluation scenarios, where for each rib only one observation was considered: the one with the lowest class assigned, the one with the modal class assigned, and the one with the highest class assigned. Modal class was defined as the most frequently observed value between the five original assignments, and the closest to the mean value in case of a tie (e.g., for the series 2, 2, 5, 7, 7, the mode was considered to be 7).

We expected that the modal class would have the most accurate rating, since it represents a balance between the two most extreme choices. Instead, to our surprise, age was estimated much more successfully when systematically considering only the highest of all observations when applying İşcan classification, raising the success rate for both operators to almost 75%; this did not happen when applying Hartnett classification. However, it must be noted that assignment unbalance (a difference of two or more classes among the same rib observations) was more prevalent in the İşcan evaluation (15 and 19 instances for each operator) than in the Hartnett evaluation (15 instances each).

The results of this sensitivity analysis are summarized in detail in Table 2.

**Table 2:** Age estimation success rate: baseline and maximization strategy results.

Operator	Baseline	Min	Max	Mode	Improvement
OP1 (İşcan)	0.63	0.53	0.74	0.62	+0.11
OP2 (İşcan)	0.60	0.44	0.75	0.61	+0.15
OP1 (Hartnett)	0.40	0.35	0.47	0.40	+0.07
OP2 (Hartnett)	0.36	0.33	0.35	0.36	−0.01

## Discussion

In this study, a sample of 127 pairs of fourth ribs was analysed. Both the right and left ribs of each individual were collected and observed, in contrast to what was observed in the work of İşcan and Hartnett, where only the fourth right rib was taken into account [6, 7, 13]. The study was not however designed to highlight any difference arising from left *vs.* right rib, as both ribs were observed simultaneously; as such, the operators were aware that both ribs belonged to the same subject. This being a potential source of classification bias when differentiating between left and right, no hypothesis was advanced on potential differences in asymmetry, which may be object of future studies.

For the İşcan method, intraoperator agreement was moderate for both operators; the interoperator agreement was also moderate (*P* < 0.001 for all measurements). In a similar study conducted by Haj Salem et al. [18] on a sample consisting of 108 fragments of the fourth rib of male Tunisian subjects, a greater intraoperator agreement was obtained; also the interoperator agreement was better.

Applying the Hartnett classification to our sample, similar agreement results were obtained.

Applying the two methods on a sample of 313 subjects, Merritt [19] obtained an intraoperator agreement of 0.870 for both (*P* < 0.001). However, it has been observed in both Merritt’s study and ours that the İşcan and Hartnett methods have a low overall success rate in assigning the correct age at death. More specifically, Merritt found that for İşcan classification the success rate was 57.5%, while for Hartnett classification the success rate was 29.7%. In our sample, the İşcan assignment success rate was found to be 62% and the Hartnett assignment success rate was found to be 38%. Thus, there is not a large percentage of success with either method. The overall success rate is lower for the revised Hartnett method, probably due to the narrowness of the age ranges.

When evaluating prior and posterior distribution densities, we expected the two curves to overlap. However, applying the İşcan method had a good overlap only for classes II, III, and IV, with no good overlap for classes V and higher (Figure 2). Applying the Hartnett method, a better overlap was observed, with notable exceptions for class II and especially class VII (Figure 3). These observations suggest that the Hartnett method is easier to use, and that the İşcan method has a greater margin of error, probably because the age ranges corresponding to classes higher than IV have a greater degree of overlap, so they tend to mislead operators.

After evaluating the reliability of the methods of İşcan and Hartnett by applying them to a sample of our direct observation, highlighting for both a low success rate, a rule of thumb has been proposed to increase the chances of obtaining a reliable estimate for the age of death. We observed that the success rate increased when, across the five repeated observations, the highest assigned class was set as the one of choice. This is especially true when applied using the İşcan method, reaching a success rate of ~75%. Such a result may be probably due to the fact that İşcan classification tends to underestimate the age at death, and presents a perfect overlap of the age ranges in the last four phases. This means that, when using the İşcan method to estimate the age at death, it is recommended to assign the highest class in case of doubt.

Applying, instead, the Hartnett classification the choice of the highest class among the five assigned in case of doubt brings a lower increase in the success rate: in our sample there was an improvement of +0.07 only for OP 1 and no improvement for OP 2, against an improvement of +0.11 and +0.15 for OP 1 and OP 2, respectively, using the İşcan method. This result is probably due to the fact that the Hartnett phases correspond to narrower and less overlapping age ranges. However, it has been seen that for this greater and better definition of the intervals of age the imbalance in the assignment of phases in the various observations is smaller for Hartnett than for İşcan.

## Conclusions

The most used methods to estimate the age in skeletal remains of adult individuals were based on morphological analysis of cranial suture closure and pubic symphysis [4].

However, several studies have shown that the morphological analysis of cranial suture closure is not a reliable method for the estimation of age since it is age-independent and influenced by sex-related phenomena [3, 20]. Similarly, it has been observed that the pubic symphysis is subject to considerable functional stress, being directly involved in supporting body weight, in locomotion, pregnancy, and childbirth, and therefore presents a great variability both between the two sexes and in subjects of the same sex. In contrast, the ribs are mostly involved in respiratory mechanics, an activity that presents minimal variations in healthy subjects [21]. For all these reasons, study of the fourth rib represents the most reliable method in the estimation of age in skeletal remains.

The method of estimation of age at death based on morphological analysis of the sternal end of the fourth rib, devised by İşcan and subsequently revised by Hartnett, represents a more reliable approach than others in the process of identifying skeletal remains. However, it has some limitations that require taking some precautions for its correct application.

First, as shown in similar studies, the reliability of the method increases with the training of the operators. However, some steps can be taken to increase both methods’ reliability, even when less experienced operators are to apply them.

The Hartnett method has a lower error rate in the procedure of assigning the ribs to the correct age stage. When using the İşcan method, instead, it is advisable to repeat the observations multiple times, as in our case, and then choose the highest class among those assigned. This increases the probability of assigning a correct age range.

## References

[1] Marella GL . Elementi di Antropologia Forense. Dalle Indagini di Sopralluogo Agli Accertamenti di Laboratorio. 1st ed. Milan (Italy): Cedam; 2003. Italian.

[2] Bonicelli A, Xhemali B, Kranioti EF, et al. Rib biomechanical properties exhibit diagnostic potential for accurate ageing in forensic investigations. PLoS One. 2017;12:e0176785. 28520764 10.1371/journal.pone.0176785 PMC5435173

[3] Franklin D . Forensic age estimation in human skeletal remains: current concepts and future directions. Leg Med (Tokyo). 2010;12:1–7. 19853490 10.1016/j.legalmed.2009.09.001

[4] Scheuer L . Application of osteology to forensic medicine. Clin Anat. 2002;15:297–312. 12112359 10.1002/ca.10028

[5] İşcan MY, Loth SR, Wright RK. Metamorphosis at the sternal rib end: a new method to estimate age at death in white males. Am J Phys Anthropol. 1984;65:147–156. 6507605 10.1002/ajpa.1330650206

[6] İşcan MY, Loth SR, Wright RK. Age estimation from the rib by phase analysis: white males. J Forensic Sci. 1984;29:1094–1104. 6502109

[7] İşcan MY, Loth SR, Wright RK. Age estimation from the rib by phase analysis: white females. J Forensic Sci. 1985;30:853–863. 4031812

[8] İşcan MY . Osteometric analysis of sexual dimorphism in the sternal end of the rib. J Forensic Sci. 1985;30:1090–1099. 4067537

[9] Sanders CF . Correspondence—sexing by costal cartilage calcification. Br J Radiol. 1966;39:233. 10.1259/0007-1285-39-459-233-a 5935201

[10] Koçak A, Özgür Aktas E, Ertürk S, et al. Sex determination from the sternal end of the rib by osteometric analysis. Leg Med (Tokyo). 2003;5:100–104. 12935539 10.1016/s1344-6223(03)00045-2

[11] Macaluso PJ, Rico A, Santos M, et al. Osteometric sex discrimination from the sternal extremity of the fourth rib in a recent forensic sample from Southwestern Spain. Forensic Sci Int. 2012;223:375.e1–375.e5. 10.1016/j.forsciint.2012.09.007 23068090

[12] Darwish RT, Abdel-Aziz MH, El Nekiedy A-AM, et al. Sex determination from chest measurements in a sample of Egyptian adults using multislice computed tomography. J Forensic Leg Med. 2017;52:154–158. 28938228 10.1016/j.jflm.2017.09.006

[13] Hartnett KM . Analysis of age-at-death estimation using data from a new, modern autopsy sample—part II: sternal end of the fourth rib. J Forensic Sci. 2010;55:1152–1156. 20456580 10.1111/j.1556-4029.2010.01415.x

[14] Rosenblatt M . Remarks on some nonparametric estimates of a density function. Ann Math Statist. 1956;27:832–837.

[15] Landis JR, Koch GG. The measurement of observer agreement for categorical data. Biometrics. 1977;33:159–174. 843571

[16] Kaplan DM . Distcomp: comparing distributions. Stata J. 2019;19:832–848.

[17] Kottner J, Audige L, Brorson S, et al. Guidelines for reporting reliability and agreement studies (GRRAS) were proposed. Int J Nurs Stud. 2011;48:661–671. 21514934 10.1016/j.ijnurstu.2011.01.016

[18] Haj Salem N, Aissaoui A, Mesrati MA, et al. Age estimation from the sternal end of the fourth rib: a study of the validity of İşcan’s method in Tunisian male population. Leg Med (Tokyo). 2014;16:385–389. 25027181 10.1016/j.legalmed.2014.06.007

[19] Merritt CE . A test of Hartnett’s revisions to the pubic symphysis and fourth rib methods on a modern sample. J Forensic Sci. 2014;59:703–711. 24602081 10.1111/1556-4029.12380

[20] Hershkovitz I, Latimer B, Dutour O, et al. Why do we fail in aging the skull from the sagittal suture? Am J Phys Anthropol. 1997;103:393–399. 9261501 10.1002/(SICI)1096-8644(199707)103:3<393::AID-AJPA8>3.0.CO;2-R

[21] Yavuz MF, İşcan MY, Çöloğlu AS. Age assessment by rib phase analysis in Turks. Forensic Sci Int. 1998;98:47–54. 10036759 10.1016/s0379-0738(98)00122-4

